# “I can’t imagine having to do it on your own”: a qualitative study on postoperative transitions in care from the perspectives of older adults with frailty

**DOI:** 10.1186/s12877-023-04576-9

**Published:** 2023-12-13

**Authors:** Emily Hladkowicz, Mohammad Auais, Gurlavine Kidd, Daniel I. McIsaac, Jordan Miller

**Affiliations:** 1 School of Rehabilitation Therapy, Queen’s University, Kingston, Canada; 2 Clinical Epidemiology Program, The Ottawa Hospital Research Institute, Ottawa, Canada; 3 Department of Anesthesiology and Pain Medicine, The Ottawa Hospital, Ottawa, Canada; 4 Patient Engagement in Research Activities, The Ottawa Hospital Research Institute, Ottawa, Canada; 5 Department of Anesthesiology and Pain Medicine, University of Ottawa, The Ottawa Hospital, Ottawa, Canada; 6 School of Epidemiology & Public Health, University of Ottawa, Ottawa, Canada

**Keywords:** Transitions in care, Frailty, Surgery, Qualitative research

## Abstract

**Background:**

Adults aged 65 and older have surgery more often than younger people and often live with frailty. The postoperative transition in care from hospital to home after surgey is a challenging time for older adults with frailty as they often experience negative outcomes. Improving postoperative transitions in care for older adults with frailty is a priority. However, little knowledge from the perspective of older adults with frailty is available to support meaningful improvements in postoperative transitions in care.

**Objective:**

To explore what is important to older adults with frailty during a postoperative transition in care.

**Methods:**

This qualitative study used an interpretive description methodology. Twelve adults aged ≥ 65 years with frailty (Clinical Frailty Scale score ≥ 4) who had an inpatient elective surgery and could speak in English participated in a telephone-based, semi-structured interview. Audio files were transcribed and analyzed using thematic analysis.

**Results:**

Five themes were constructed: 1) valuing going home after surgery; 2) feeling empowered through knowledge and resources; 3) focusing on medical and functional recovery; 4) informal caregivers and family members play multiple integral roles; and 5) feeling supported by healthcare providers through continuity of care. Each theme had 3 sub-themes.

**Conclusion:**

Future programs should focus on supporting patients to return home by empowering patients with resources and clear communication, ensuring continuity of care, creating access to homecare and virtual support, focusing on functional and medical recovery, and recognizing the invaluable role of informal caregivers.

**Supplementary Information:**

The online version contains supplementary material available at 10.1186/s12877-023-04576-9.

## Introduction

Transitional care is defined as “a set of actions designed to ensure the coordination and continuity of health care as patients transfer between different locations or different levels of care within the same location” [1]. Following elective surgery, the transition from hospital to home is a difficult time that can negatively affect patient [2], and healthcare system-level outcomes [3]. This is particulary true for older adults with frailty [4–7], a state that contributes to an increased vulnerability to stressors leading to adverse health outcomes [8, 9]. As older adults are having elective surgery at an increasing rate [10], and given the challenges and risks they face during the transition home after surgery, there is an urgent need to improve this transition in care [11]. To provide meaningful, patient-centered improvements in care for this vulnerable population, it is important to first gain insight into what is important to older adults with frailty as they experience a postoperative transition in care.

Poor quality transitions in care can lead to medical errors [12], adverse drug events [13, 14], Emergency Department (ED) visits [15], readmission [16] and unmet patient needs [17]. These risks become heightened for older adults with frailty, who are already at an increased risk of negative outcomes following surgery. Frailty before surgery is associated with a two-fold or greater increase in adverse postoperative outcomes, including prolonged length of hospital stay [4], complications [4], readmission [18], new patient-reported disability [5], non-home discharge [19], and death [20, 21]. Therefore, is is not surprising that following elective surgery, older adults with frailty require the most support during their postoperative transition in care [22].

A body of literature on the transition from hospital to home for older adults exists. One scoping review of transitional care interventions to support older adults moving from hospital to home identified that readmission and mortality were the most common outcomes used to evaluate these transitions [23], which may not be the most important outcomes from the perspective of patients [24]. Further, none of the articles in the scoping review included older surgical patients [23]. A systematic review and meta-analysis of transitional care interventions for older adults with frailty [25] discovered variability in intervention components, which healthcare providers were involved, and what outcomes were used to evaluate the transition in care. While the review had a broad inclusion criteria, only 1 of 21 articles included older adults with frailty having surgery, and only 16% of the study sample had surgery [25]. Finally, one scoping review specific to transitions in care after surgery for older adults found substantial variability in how the quality and success of postoperative transitions are evaluated [26], only two articles in the scoping review included frailty assessments [27, 28], and no study explored the experiences of the transition in care after surgery from the perspective of older adults with frailty. Notably, none of the included articles engaged patients with lived experience in their research. It is clear that innovative interventions need to be developed to improve patient-centered outcomes for older adults with frailty experiencing a postoperative transition in care from hospital to home.

Before developing and evaluating postoperative transitional care interventions for older adults with frailty, it is important to consider the unique, and added challenges of returning home after elective surgery for older adults living with frailty, who experience a 2-fold increase in new patient-reported disability after surgery, and a 5-fold decrease in successful hospital to home discharge [5]. One meta-summary of qualitative findings exploring the hospital-to-home transition for older adults highlighted that patients experience an insecure and unsafe transition, and that there is a need for addressing several missing components including assessment and planning before going home, engagement of patients and informal caregivers, care coordination and supporting self-management [29]. Notably, the majority of the 13 included articles focused on non-surgical patients, and of the studies that did explore the postoperative transition in care, none included older adults with frailty [29].

While there is an increasing body of qualitative literature on transitions in care from the perspectives of older adults including in the context of transitions following non-surgical admissions [30], hip fracture surgery [31], and at end of life [32], to our knowledge and based on the recent reviews highlighted, there remains a gap in understanding the experience of older adults with frailty after surgery.

Elective surgery is one of the only hospital admissions that is planned for in advance, meaning that patients, caregivers, providers and the healthcare system can also prepare for the postoperative transition in care. Gaining insights into the perspectives of older adults as they transition from hospital to home after surgery represents an important step forward in improving transitions in care. Therefore, the purpose of this study was to understand what is important to older adults with frailty during a postoperative transition in care. This research will contribute to the patient-centered literature on postoperative transitions in care by addressing the current gap of the qualitative study of older adults with frailty experiencing a transition in care after surgery. By understanding what is important to older adults with frailty as they transition home after surgery, researchers, clinicians and policy-makers can begin to develop and evaluate postoperative transitional care interventions that result in meanginful improvements in care based on the preferences and needs of patients.

## Materials & methods

### Study design

This was an interpretive description qualitative study [33, 34] using semi-structured interviews to explore what is important to older adults with frailty during a postoperative transition in care. As transitions in care require individual and pragmatic considerations, interpretive description was chosen because this methodology was well-aligned with our goal of generating new knowledge that can be applied in real-world settings [33, 34]. Interpretive description involves using an analytic framework and rigor in the research process to identify results that can be applied in healthcare settings, while acknowledging that new concepts and varying contexts can re-shape how the results are interpreted and considered beyond the initial analytic framework [33, 34]. The ontological and epistemological underpinnings are situated within a constructivist paradigm [35] which conceives that there are multiple realities and truths, and that pieces of knowledge are situated in meaning and interpretation [35, 36]. The reflexive role of the researcher is an integral aspect of this qualitive methodology [36]. For the purpose of this research, reflexivity was defined as the researcher critically reflecting and acknowledging their role and situatedness in all phases of the research study and how this might affect the research process, including how knowledge is developed and shared [37, 38]. Practicing reflexivity means that the researcher must “interrogate” their own positions and “locate” how their perspectives, values, assumptions, etc., shape the research process [36, 39]. The study was conducted and reported in alignment with the Consolidated Criteria for Reporting Qualitative Research checklist (COREQ) [40].

### Setting and participants

This study was conducted at a multicentre academic health sciences network, The Ottawa Hospital (TOH), which provides inpatient surgical care at two of its campuses (Civic and General sites) to a catchment area of 1.2 million people in Ottawa, Canada. TOH has 900 inpatient beds, and is the regional referral center for trauma, vascular, neuro, thoracic and complex oncology surgery.

Individuals were invited to participate if they met the following criteria: 1) ≥ 65 years of age on the date of surgery, 2) had an inpatient, elective, non-cardiac surgery, 3) a pre-operative Clinical Frailty Scale (CFS) score of ≥ 4,^9^ and 4) were able to complete the interview in English. Eligible participants who provided their consent to be approached for research were screened for eligibility. EH reviewed the patient’s chart to determine the pre-operative CFS score. Research has shown that CFS scores can be reliably generated by chart review and is consistent with a patient interview [41]. A purposive sampling strategy [42] was used to identify potential participants to contact. We enrolled similar number of males and females, participants of various ages, and different surgery types. Prior to participation, participants provided verbal consent over the telephone. Recruitment and data collection ended once information power [43] was achieved which was assessed by evaluating the study aim, sample specificity, quality of the dialogue and analytic technique [43]. The authors (EH, JM) evaluated these components as data collection and analysis continued and recruitment ended once the authors felt that there was quality and comprehensive data to support the research question.

### Ethical considerations

Ethics approval was obtained from The Ottawa Health Sciences Network – Research Ethics Board (Protocol# 20200322-01H) and Queen’s University Health Sciences & Affiliated Teaching Hospitals Research Ethics Board (Protocol# REH-773–20) prior to the commencement of participant recruitment and data collection. Particpants provided verbal informed consent before participating in the study. Participants were not provided any compensation for their participation as data collection was done over the telephone at their own convenience. Participants were informed that their participation in the study was voluntary and that their information would be kept confidential. As such, only de-identified quotes where anonymity is preserved, will be shared upon request.

### Data collection

Demographic information was collected using a telephone-based questionnaire. Medical data, including comorbidities, in-hospital postoperative complications and medication count at admission versus discharge were collected through an electronic medical record review by a research assistant. Individual telephone interviews were conducted using a semi-structured interview guide (Additional File 1). Interviews were conducted within one year following surgery. The overall structure of the interview guide was informed by a continuum of care framework [44] that highlights the transition points for a hospitalization (admission to hospital, hospital stay and discharge planning, transition out of hospital) to help participants recall their journey and to highlight what was important to them, particularly during the postoperative transition in care. The interview guide was reviewed and revised by a patient partner (GK). All interviews were conducted by EH. The average length of the interview was 42 min. During the informed consent process and the interview, participants were reminded that they could either take a break or stop the interview at any point. EH engaged in reflexive journalling before and after each interview (taking note of emotions and the meaning of words used by participants), took field notes during the interviews, and continued to journal throughout the analytic process.

### Analysis

Interviews were audio-recorded, transcribed, reviewed for accuracy and then analyzed using reflexive thematic analysis [36, 45], in an interpretive description tradition [34], to identify clinically meaningful themes. A combination of coding on hardcopy transcripts and using NVivo 12 Pro software was used for organizing data within codes, themes and subthemes.

While flexibility with the analytic process is a distinctive feature of reflexive thematic analysis, the following phases were applied and followed [36, 39]: (1) dataset familiarization; (2) data coding; (3) initial theme generation; (4) theme development and review; (5) theme refining, defining and naming; and (6) writing up. Two study investigators (EH, JM) independently coded the first two transcripts and met to discuss, and critically reflect upon their codes, to generate creativity which supported ongoing coding. As recommended in reflexive thematic analysis, the goal of engaging two coders was not to reach consensus but to develop deeper and more insightful analysis [36]. For the remaining transcripts, the lead author (EH) conducted the data coding [36, 39]. Once codes were identified a coding tree was developed (Additional File 2).

### Credibility and reflexivity

In alignment with interpretive description, credibility is persevered when the study purpose, process and context is made clear [33, 34]. The purpose of the study was to explore what is important to older adults with frailty during a postoperative transition in care. The process of data collection and analysis was detailed in the form of an audit trial, documenting analytic notes and decisions regarding themes and sub-themes. Further, the research team met to discuss the data in the form of a “sounding board” to encourage reflexivity [46].

#### Reflexivity statement

EH, the author who led data collection and analysis, is a Doctoral candidate in an Aging & Health Program, works as a Research Associate in a perioperative research program and has completed a frailty fellowship through the Canadian Frailty Network (for a full reflexivity statement for EH, see Additional File 3). MA is an Assistant Professor in the School of Rehabilitation Therapy and is a registered physical therapist with expertise in geriatric rehabilitation. GK is a patient partner with lived experience as an older adult who has had inpatient elective surgery and is a retired Social Worker. DIM is an anesthesiologist and scientisit who conducts clinical trials to improve patient and system-level outcomes of older people having surgery. JM is a physiotherapist and Assistant Professor in the School of Rehabilitation and conducts quantitative and qualitative health services, pain management, and primary care research. None of the investigators had existing relationships with any of the participants.

## Results

### Participants and characteristics

Demographic and transitions in care details are in Table 1. None of the participants experienced a readmission within 30 days of surgery, one visited the Emergency Department (ED), and all participants had a follow-up (virtual or in-person) with their surgeon within 60 days of surgery. Only one participant had the same number of medications at admission and discharge whereas the other 11 participants all had more medications at time of discharge. Of the 12 participants, five had documentation of home care services in their discharge notes (two were documentation of new referrals, and three were confirmation of homecare in place). Half of the sample had surgery before the COVID-19 pandemic was declared and half had surgery after the COVID-19 pandemic was declared (surgery dates ranged between December 2019 – March 2022). All interviews were conducted during the COVID-19 pandemic.

**Table 1:** Participant demographics and transition in care details

Mean age (range)	76 (65–85)	
Gender, n (%)	Man – 6 (50)	
	Woman – 6 (50)	
CFS score, n (%)	4 [vulnerable] – 6 (50)	
	5 [mildly frail] – 6 (50)	
Hospital length of stay, n (%)	1–2 days – 1 (8)	7–8 days – 1 (8)
	3–4 days – 5 (41)	29 days – 1 (8)
	5–6 days – 4 (33)	
Surgical specialty, n (%)	Thoracic – 3 (25)	
	Urology – 3 (25)	
	General – 2 (17)	
	Gyne-oncology – 1 (8)	
	Vascular – 2 (17)	
	Otolaryngology – 1 (8)	
Number of comorbidities, n (%)	1–5 comorbidities—6 (50)	
	6–10 comorbidities—3 (25)	
	11–15 comorbidities—3 (25)	
Clavien Dindo classification of postoperative complications, n (%) (this tool is used to classify the severity of postoperative surgical complications) [47]	None—4 (33.5)	
	Mild—3 (25)	
	Moderate—4 (33.5)	
	Severe—1 (8)	
Caregiver support, n (%)	Spouse – 7 (58)	
	Adult child (living separately) – 4 (33)	
	Adult child (living together) – 1 (8)	
Time between sugery and interview, n (%)	3 months – 2 (16) 7 months – 1 (8)	
	4 months – 2 (16) 9 months – 4 (33)	
	5 months – 2 (16) 10 months – 1 (8)	
Ethnicity, n (%)	White – 12 (100)	
Highest education level, n (%)	Primary school or less – 1 (8)	
	Some high school – 1 (8)	
	Completed high school – 4 (33)	
	Some college/university but did not finish – 2 (17)	
	Undergraduate diploma/degree from college/university – 2 (17)	
	Graduate school – 2 (17)	

### Themes

**Fig. 1:**
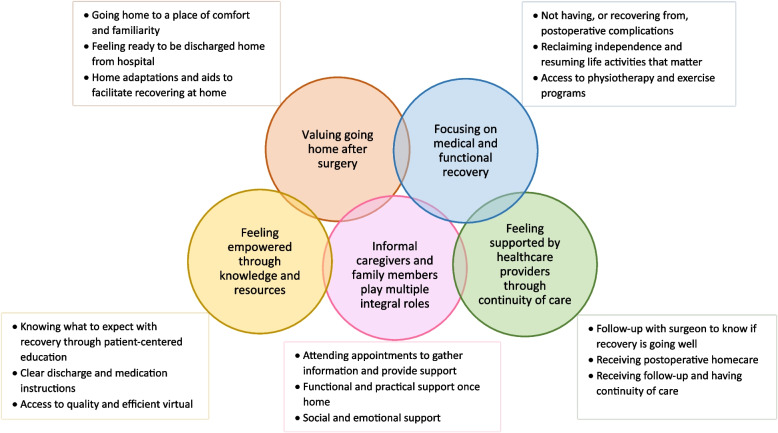
Themes and sub-themes

Five themes were constructed from the exploration of what is important to older adults with frailty during their postoperative transition in care: 1) valuing going home after surgery; 2) feeling empowered through knowledge and resources; 3) focusing on medical and functional recovery; 4) informal caregivers and family members play multiple integral roles; and 5) feeling supported by healthcare providers through continuity of care. Each theme has 3 sub-themes (Fig. 1).

### Theme 1: valuing going home after surgery

Home signified normalcy, comfort and healing. It was important that participants felt ready to go home, with some requiring home adaptations or functional aids to do so.

#### Sub-theme 1: going home to a place of comfort and familiarity

One participant shared that being at home facilitated improvements in quality of life and that being home was a sign of healing:

*A very happy feeling [to be home]. It makes a huge difference. You know, if you’re used to your home, it’s very important … your quality of life just goes up… I can just see a big relief, you know? In a hospital setting, you know you’re sick … when you’re home, you think you’re on your way of progress, you know? T-009*

#### Sub-theme 2: feeling ready to be discharged home from hospital

While participants were eager to get home, it was important to them that they felt ready and well enough to go home. One participant expressed:

*And it was just the day before I went home … she said, "Well get dressed because you're going to be picked up at such and such a time." So anyway, I said, "Okay." But I said, "I haven't really, you know, I haven't really walked." T-007*

#### Sub-theme 3: home adaptations and aids to facilitate recovery at home

To facilitate being home, participants highlighted the importance of either adapting aspects of their home environment or using functional aids. One participant shared:

*They had advised me that maybe I'd be comfortable for the first little while if I slept in a La-Z-Boy with all the tubes and stuff. You know, I might find it easier. So, we did - my daughter went to Canadian Medical and I rented a La-Z-Boy. T-004*

### Theme 2: feeling empowered through knowledge and resources

Participants wanted to be provided with clear and relevant information from healthcare providers. Participants wanted to have the knowledge to know what to expect so they could make informed decisions.

#### Sub-theme 1: knowing what to expect with recovery through patient-centered education

Participants wanted to receive information during their preoperative appointments and at hospital discharge that aligned with their values and goals. One participant shared:

*They told me what to do with the drip bag and that the CCAC would be coming in to check the dressing around the drip bag, that was about it … if I was more aware of what it would be like as far as pain or my mentality…that would have been helpful. Well I’d want to know— what I’m going to feel like? What’s my body going to feel like? What can’t I do? What can I do? T-012*

#### Sub-theme 2: clear discharge and medication instructions

Participants described being provided with a lot of information during their perioperative journey and wanted clear discharge instructions. One participant expressed:

*I think number one would be the clear explanation of medications and follow-up expectations. Number two would be a clear set of instructions as to what to do once I got home for both my wife and myself. And number three would be if I were to have to have dressings changed, for example, to make sure that was set up. T-013*

#### Sub-theme 3: access to quality and efficient virtual support

Participants shared concern about not knowing who to call if they had important questions that might require immediate attention. One participant voiced:

*You know, we get all this information like who you should call when you have a problem and one of them is Telehealth, Ontario. For instance, a little while back we were concerned about something, we phoned them. Well - and we've done this a couple of times now - it could be eight to ten hours before somebody, a nurse would be able to get back to us. Can you believe it? She said, "If you're worried, go to the emergency." Nobody can talk to you - well like this is just the receptionist, of course she can't give you any information other than to tell you the nurse will not be available for eight to ten hours. By that time, you'll be dead for sure. T-003*

### Theme 3: Focusing on medical and functional recovery

Participants wanted to avoid postoperative complications and wanted to be able to re-join life activities that matter to them.

#### Sub-theme 1: not having, or recovering from, postoperative complications

Some participants experienced postoperative complications while others did not. For those who didn’t, they felt very thankful that they didn’t have any complications and described that as being very important. One participant expressed:

*It wasn’t expected, obviously. But, given that I had to have a drain tube in almost longer than anybody in recorded history apparently… that wasn’t an experience that I looked forward to, let’s put it that way. And it was a certain measure of discomfort and misery … it affected my recovery because it was much slower than expected. T-011*

#### Sub-theme 2: reclaiming independence and resuming life activities that matter

Participants described the importance of outcomes that extend beyond medical outcomes, such as returning to their independence, and resuming meaningful life activities. One participant said:

*You like to be able to get out of your house and get out and walk around and do things. Like to go shopping … go to restaurants, out to visit friends. And I’ve got a soon-to-be six-year-old grandson, who…requires a lot of help in playing with dinosaurs. So, you know, you want to be able to kick the soccer ball around and you want to be able to do things like that and, you know … those are important life things that you like to be able to do. And you kind of hope that you’re going to recover from the surgery just to be well that you can do all of those things. T-012*

#### Sub-theme 3: access to physiotherapy and exercise programs

Participants desired postoperative physiotherapy and exercise. However, some mentioned not being informed about these services. One participant reflected:

*And I think like if I’d known about something like [an exercise class] at the beginning and my doctor said, “Okay, yeah you can try it,” I would have gone. And I would be in better shape today. And that’s why I think like if they could arrange with the physiotherapy centre at the hospital. You know? That I go there and they show me what exercises to do. Do them, or get on a treadmill or whatever and said, “Okay, go home and do this, this and this every day.” And I think it would have made my life a lot better. I would be in a lot better shape today. T-012*

### Theme 4: Informal caregivers and family members play multiple integral roles

Participants described the necessity of having multiple people involved in supporting their postoperative transition in care, including their spouse, adult children, other family and neighbours.

#### Sub-theme 1: attending appointments to gather information and provide support

Having a support person present allowed participants to have a second set of ears to listen to all of the information. Some caregivers received training on medical supports. One participant described:

*They were able to bring my wife in to get some training on the drip machine for the feeding tube … so my wife was able to get really trained on that. The actual home care folks didn't have a lot of experience with that feeding tube system we had ... my wife was able to pick up everything from the hospital … I was pretty fuzzy in my head and I couldn't have operated that myself. T-009*

#### Sub-theme 2: functional and practical support once home

Participants expressed needing help with activities like bathing and getting to appointments. One participant described:

*I needed the help. I needed the help for [showering and getting dressed]. Well I couldn’t take a shower or bath because of my surgery … Except for I could get up and walk to the bathroom, go to the bathroom. I was fine with that, but it was nice to have [my daughter(s)] to reassure me. T-006*

One participant explained how they are typically a caregiver to their husband but needed the support of their daughter to care for him so she could recover from her surgery:

*[My daughter] looked after her father … Sometimes throughout the night time I have to get up with him to go to the washroom … my daughter keeps offering. She says, "Mom, why don't you take a break and come - you take my room tonight and I'll go in and sleep in your bed so you can get a break." ... It's just that we're afraid of falls with him because he had several, you know? T-002.*

#### Sub-theme 3: social and emotional support

Participants gained invaluable emotional strength through their connections and relationships. One participant said:

*I've got an understanding family that looked after my every need … I think you get a lot of strength and support from your family. I can't imagine having to do it on your own … looking after yourself … it would be major because you don't have a lot of strength and I'm sure there would be many tearful moments at home on my own. T-010*

### Theme 5: feeling supported by healthcare providers through continuity of care

Participants wanted to feel supported by their healthcare team and receive follow-up appointments and continuity of care from their surgeon, homecare workers, allied health professionals and family doctor.

#### Sub-theme 1: follow-up with surgeon to know if recovery is going well

Seeing their surgeon after surgery was important for participants as they knew that their surgeon would be able to confirm whether they were healing, as explained by the following participant:

*I had a meeting with the surgeon who did my surgery. I had a physical meeting with him a month after the surgery … I think it gives you the confidence that things are progressing well. T-009*

#### Sub-theme 2: receiving postoperative homecare

Participants expressed their reliance on homecare for their medical care during such a vulnerable time. One participant shared:

*The nutritionist [that came to my home] was excellent. She also was a former nurse, so she had a really good understanding of this and she provided a lot of the information that I think we were looking for … and she was very engaged … which was [important] when you're completely vulnerable, which I was ... she came once a week. Also contact by phone, so we had steady contact with her … We [also] had three nurses come in … making sure that - they're in the field and they are current with everything, so if there was anything that was going wrong, they would make me aware of - my wife aware of what, what needs to be done, you know? That's why you have special nurses. T-009*

#### Sub-theme 3: receiving follow-up and having continuity of care

Many participants valued knowing their family doctor would be available for support. Others expressed wanting a known healthcare provider to check-in on them. One participant expressed:

*It would be nice if a social worker came to see me in the hospital and it would be nice if that same person followed-up with me at home, whether it be online or by phone, or come to the house. T-001*

## Discussion

In this qualitative study of older adults with frailty who experienced a postoperative transition in care, five themes highlight what is important to patients: 1) valuing going home after surgery, 2) feeling empowered through knowledge and resources, 3) focusing on medical and functional recovery, 4) informal caregivers and family members play multiple integral roles and 5) feeling supported by healthcare providers through continuity of care. These findings can inform improvements to the perioperative journey (preoperative, in-hospital, postoperative) that will support the postoperative transition in care for older adults with frailty having elective surgery. Researchers, clinicians and policy makers should consider these elements, informed by patients' voices, when developing, evaluating and implementing transitional care interventions for older adults with frailty after surgery.

Feeling empowered throughout all stages of the perioperative journey was important to prepare participants for the period after surgery. While there are various definitions of empowerment, patient empowerment can occur when patients are encouraged to be involved in their decisions and supported in the management of their care [48]. To support decision-making, patients require information [49]. Participants in the current study wanted to know what to expect after surgery through patient-centered education, beginning in the preoperative period. They wanted to know what to expect during their recovery based on what was important to them (i.e., functional limitations, level of functional independence, pain, mental health, etc.). This finding aligns with, and builds upon, a large body of evidence on the importance of providing meaningful information about what to expect after surgery in a way that is understandable [50, 51]. Further, several participants highlighted the need to have home adaptations, functional aids, and access to physiotherapy or exercise supports as important strategies to support their postoperative transition in care. Discussing these needs and how to access these supports at the preoperative phase rather than at discharge could alleviate stress for patients and their caregivers who often find it difficult to coordinate once home. While provision of information does not necessarily yield empowerment or improved outcomes, patient-centered information, provided in a clear and understandtable way, could support patient decision-making, planning, and self-management strategies to support the postoperative transition in care. Future research is needed to evaluate the most useful ways for presenting this key information to patients.

Participants in this study identified a need to focus on their functional recovery in addition to medical recovery. They placed emphasis on the importance of avoiding or recovering from complications and resuming life activities that matter. This is consistent with other research which found that older adults value a return to recreational activities, mobility and activities of every day living after surgery [52]. The current study indicates that older adults desire information about, and access to, postoperative physiotherapy and exercise programming to support achievement of these functional outccomes that participants valued. Clinicians and policy makers should work together to ensure that patients are informed about, and have access to, physiotherapy and exercise programming to support these key functional outcomes.

Previous research has shown that older adults having elective surgery identify going home as an important outcome [53]. While going home was highlighted in the current study, it was also important for participants to feel ready to go home. This is in line with a previous qualitative study of older adults having hip replacement surgery that found it was important for patients to feel confident, safe and to have support of family and friends to feel ready to be discharged home [54]. Participants in the currenty study identified the importance of opportunities to walk around the hospital to feel more confident in their readiness to go home. Additionally, participants wanted their pain controlled and their symptoms managed before being discharged, and wanted to feel confident (and/or their informal caregivers to feel confident) that they had the knowledge and skills to manage their care at home. Effective communication and patient/caregiver education in the hospital setting could potentially be a way of increasing confidence with readiness for discharge, however more research in this area is needed. Importantly, participants wanted communication and instructions to be clear and informative for them and their informal caregivers. Previous research on transitions in care has similarly highlighted that older adults see a need for improved communication and that instructions could be clearer with simpler language [30]. Further, previous research has demonstrated that effective communication needs to consider patient preferences, individual contexts and changes in health [55], and needs to be maintained after follow-up to support continuity of care [30].

Participants expressed concern over monitoring their postoperative symptoms at home and wanted efficient virtual support for the postoperative period at home. One emerging technique for monitoring patient symptoms after surgery is through virtual recovery after surgery (VRAS) techniques such as remote automated monitoring (RAM) where biophysical variables (i.e. blood pressure) can be captured through technology and monitored by clinicians [56, 57]. There is promising evidence to suggest feasibility and patient satisfaction with this type of virtual symptom monitoring after surgery [58]. The impact on outcomes is sparse, yet encouraging, with one randomized controlled trial showing fewer participants experiencing pain and more drug errors detected and corrected for those who received RAM [57]. Future research is needed to explore the cost and feasibility of VRAS interventions for older adults after surgery and their caregivers, and how to support patients who have higher degrees of frailty and cognitive impairment.

It has been reported that older patients who return home after a hospital admission underestimate the challenge of resuming self-care, which can result in a need for more support from caregivers [59], which has been found to affect their sense of independence [59]. Several participants in the current study shared that the emotional and physical challenges cannot be fully appreciated until it is experienced. The need for multiple caregivers and family members to support the postoperative transition in care was expressed due to the various roles that need to be filled. Our research adds rich qualitative descriptions that build on previous studies indicating that informal caregivers may be the reason why some older adults require fewer homecare services and experience a lower likelihood of transitioning into long-term care [60]. For example, one study found that patients with carers at home were more likely to stay at home and participate in a community-based transitional care program [61]. There is a need for clinicians and policy-makers to provide support to these integral informal caregivers through education, ongoing engagement and resources required to provide this challenging and necessary care.

It is encouraging that existing transitional care programs [61] include some of the elements highlighted in the current study, including optimizing patients’ home environments, facilitating access to physical therapy, and provisison of social support. However, transitional care interventions emphasize the role of informal caregivers, which is also highlighted in this study. Future research is needed to explore the experiences of older adults without a caregiver who are returning home after surgery, to understand the unique challenges they may face and how their transition in care can be supported.

Participants in our study emphasized the need for follow-up visits and homecare to support continuity of care. With older adults valuing ‘ageing-in-place’ [62], home care delivery in Europe is beginning to focus on specialized training of home care providers to teach them how to support independence and function for their patients [63]. Unfortunately, home care access in North America is limited and associated with inequities [64–66]. One Canadian study sought to understand the perspectives of 700 patients who had experienced a hospital-to-home transition and found that patients prioritized the need to improve the availability of publicly funded homecare [67]. In the current study, one of the participants recovering from surgery was also a caregiver to their aging spouse, and both could have benefited largely from homecare support. While publicly-funded homecare comes at a cost to the government, some research suggests that it is less costly to live at home with support (e.g. from a post-acute transitional care program) than to transition to a non-home location, even for older adults living with frailty [68]. Canadian policy-makers should consider the unique and heightened homecare needs of older adults with frailty having surgery.

This study used interpretive description to describe what is important to older adults with frailty during a postoperative transitions in care. Clinicians and policy makers should draw from these findings to improve how patients are informed about what to expect and how to prepare for recovery from surgery based on individual contexts and preferences, and should recognize and support the role of informal caregivers. This research also highlights that there is a need for researchers to develop and evaluate postoperative transitional care interventions for older adults with frailty, focusing on continuity of care and virtual support.

### Strengths and limitations

This qualitative study should be reviewed in the context of its strengths and limitations. While a purposive sampling strategy was used, all participants identified having at least one informal caregiver. Therefore, older adults with frailty without a caregiver were not included in this study. This may have been partly explained by the fact that people who do not have a caregiver are more likely to transition to a non-home location. Future research aimed at understanding the experiences and perspectives of older adults transitioning home after surgery from varied ethnicities, different geogprahic locations (rural verus urban) and those without a caregiver would provide valuable additional knowledge. While participants described feeling confident in recalling their experience, some of the interviews took place several months following surgery, which may have affected the recollection of some details. Further, while participants were recruited at two different hospitals, they were from within the same city, which may limit the transferability of our findings. Future work should consider the experiences of those living in other jurisdictions.

## Conclusion

The postoperative transition in care for older adults with frailty is a vulnerable time. This qualitative study provides researchers, clinicians, and policy makers with important evidence on what is important to older adults with frailty who are transitioning home after surgery. This evidence should support the future development of interventions that are aligned with the needs of patients.

### Supplementary Information

**Additional file 1.**

**Additional file 2.**

**Additional file 3.**

## Data Availability

All data generated or analysed during this study are included in this published article [and its Supplementary information files]. Any additional data are available from the corresponding author on reasonable request.

## Abbreviations

CFS: Clinical Frailty Scale
COREQ: Consolidated Criteria for Reporting Qualitative Research checklist
CQGS: Coalition for Quality in Geriatric Surgery
ED: Emergency Department
TOH: The Ottawa Hospital
RAM: Remote Automated Monitoring
VRAS: Virtual Recovery After Surgery
