# Supplementary material for: “I can’t imagine having to do it on your own”: a qualitative study on postoperative transitions in care from the perspectives of older adults with frailty
Source: BMC Geriatr. 2023 Dec 13;23:848. doi: 10.1186/s12877-023-04576-9 (PMC10716948; doi:10.1186/s12877-023-04576-9)
Supplement: Supplementary file 1 — Additional file 1. [file 12877_2023_4576_MOESM1_ESM.docx]

**Supplemental Material 1 - Interview Guide**

*Hello,*

*Thank you for agreeing to participate in this telephone interview. You have experienced going from hospital to home, which is what we call a “postoperative transition in care”. A transition in care is defined as, ‘a set of actions designed to ensure the coordination and continuity of healthcare as patients transfer between different locations”. I am really excited to speak to you because there are so many things that we often miss. The purpose of this interview is to understand what was important during the postoperative transition in care process for you and how you would describe a successful postoperative transition in care.*

1. Can you please start by describing your overall experience of your going home after surgery?
2. What happened before surgery that helped your going home?

- What did you find most important? Was there something that stood out?
- What would have been important? Was there something missing?

1. What happened in the hospital after your surgery that helped your prepare for your transition home?

- What was most important to you during your hospitalization to prepare you for your transition home? (i.e. people, resources, etc.)
- Was there anything missing / things that could have been improved that would have been helpful? What would have been important?

1. What was the discharge planning process like, and what did you find most important to you?

- Were you involved in the discharge planning? Would this be important to you?
- Were discharge instructions provided and explained to you? Were they clear?
- Was here anything missing / things that could have been improved that would have been helpful and important to you?

1. I am going to start by asking you some questions about your first few days at home. Can you describe your first few days at home.

- Was there anything missing that stood out to you that would have been good?
- What was it like for you? Did you have caregiver support?
- What was helpful for your transition home?
- What was important to you during the first few days at home?
- What was challenging about your transition home?
- What could have been improved? Was there anything missing?

1. Can you please explain any other services you required/looked out once you were discharged home?

- Did you see your family doctor for follow-up?
- Did you see your surgeon at follow up? Was it booked with your surgeon?
- Did you have home-care services in place? Was it easy?
- What was important for your transition once you were home?
- Was there anything missing once you were home that would have been be important?

1. If you were to experience another postoperative transition in care (having surgery again and going from hospital to home), what would be important to you during the transition (i.e., clear discharge education, early follow-up with GP, caregiver involvement, etc.)?
2. Would you say that your postoperative transition in care was successful? If yes, what made it successful?
3. If no, what would make a postoperative transition in care successful in your mind?
4. Is there anything else you would like to add to help me understand what was/would be important to you during a transition home after surgery?
5. Is there anything else you would like to add to help me understand what it would mean, and how I would know, that you have had a successful transition home after surgery?

Thank you so much for your time.
