# Supplementary material for: “I can’t imagine having to do it on your own”: a qualitative study on postoperative transitions in care from the perspectives of older adults with frailty
Source: BMC Geriatr. 2023 Dec 13;23:848. doi: 10.1186/s12877-023-04576-9 (PMC10716948; doi:10.1186/s12877-023-04576-9)
Supplement: Supplementary file 2 — Additional file 2. [file 12877_2023_4576_MOESM2_ESM.docx]

Supplemental Material 2 - Patient decision tree

| **Theme 1: value going home after surgery** | | | |
| --- | --- | --- | --- |
| **Sub-theme** | **Code** | **Description** | **Quotes** |
| **Sub-theme 1:** going home to a place of comfort and familiarity | Happy to go home after surgery | Feeling happy to get discharged home after surgery and to be back in their own home. | *I would have been very upset. I would have thought I was a whole lot sicker and in worse shape than I actually was. I would have thought that I was in terrible shape if I was not able to handle the surgery and go home and stay home.* |
|  | Feeling more comfortable at home | Feeling more comfortable and relaxed in their own home environment than anywhere else. | *I was ecstatic to go home … I missed my bed and I missed my wife beside me and, you know, my thing—my things around me … I love my home. I’m more comfortable. I’m more relaxed.* |
| **Sub-theme 2:** feeling ready to be discharged home from hospital | Feeling physically well to go home | Feeling well enough physically to go home, including relief of symptoms. | *Like I was very much in pain and, um, they sent me [home] … um, I had to get some, ah, pain killers …. I just, like I did not feel, um, that I was not ready to come home and, ah, I had found that hospital did not, you know, didn't keep me in long enough.* |
|  | Functionally ready to go home | Feeling confident in functional abilities to go home, including walking independently. | *And it was just the day - just before I went home. Like I, I was getting - she said, "Well get dressed because you're, you know, you're going to be picked up at such and such a time." So anyway, I said, "Okay." But I said, "I haven't really, you know, I haven't really walked." Like all I walked, it was around the room and like I just started walking, I think, the night before.* |
| **Sub-theme 3:** home adaptations and aids to facilitate recovering at home | Making adaptations at home | Adapting the home environment to be comfortable and to be able to recover at home. | *I made my own, I, ah, I modified chairs. I, ah, modified my bed. Um, a hospital bed would have been really handy, one that you can jack up, you know? So I made my own. Ah, it wasn't pretty but it, it helped.* |
|  | Devices to support living at home | Functional aids to support activities of every day living, including showering, walking safely, getting upstairs. | *And then if I got in the shower [at home] that somebody should watch me and I was very kind of scared and worried about being in the shower. But my husband went out and bought me like one of those chairs that you sit on there.* |
| **Theme 2: Feeling empowered through knowledge and resources** | | | |
| **Sub-theme** | **Code** | **Description** | **Quotes** |
| **Sub-theme 1:** knowing what to expect with recovery through patient-centered education | Clinicians explaining what to expect in a way that is understood | Clinicians using clear language to properly explain what patients can expect with surgery and their recovery. | *The surgeon himself that's what really helped me. Um, her was so positive … I was just very confident because of the way he was … they told me what to, you know what to anticipate and, ah, every time I saw them I just got more and more confident … they explained everything ... they had me well-prepared of what, ah, what - what's in store and it's going to be long.* |
|  | Providing patients with information that is meaningful and important to them | Clinicians providing benefits, risks and what to expect with recovery with examples that are important to the patients so they can make an informed decision. | *You want to know what's going on and what are the risks and what you're, you know, you could accept or refuse … I like to be informed and it's important for me not to walk into something that I don't know of.* |
|  |  |  | *In my case I couldn't imagine what was coming after. I just, I just couldn’t imagine that. I'd never had any major surgery like that before. So it's pretty hard to imagine what comes after. It was definitely beyond my realm of reality. But I knew the risks and everything. They were quite clear and I was able to prepare my entire legal aspects at home, a will and all that, which I had, but I updated everything ... which is all partial to being prepared for a major surgery like this.* |
| **Sub-theme 2:** clear discharge and medication instructions | Discharge process, information, instructions and papers | Providing patients with an information booklet at discharge and communicating clearly about what to expect over the next few weeks. | *It was efficient and sometimes it comes out a bit too fast, but if, if they fit in a little booklet along with it that's probably more helpful … especially for old people. Most of you young guys speak too fast to start with.* |
|  | Instructions on medications including what they do and when to take them | Clinicians taking the time to explain changes in medication, what the purpose of the medications are, why they are taking them, and how and when to take them. | *Well I guess I could have probably had more knowledge on the pills they were giving me … there's not instructions given to you with all the different pills …. So more instructions on what all these pills will do for you … I wanted to know what all these things do ... having somebody really explain to you is important.* |
| **Sub-theme 3:** access to quality and efficient virtual support | Knowledge of when and who to phone for support after surgery | Patients are unsure of who to contact after surgery if they have questions or concerns so it is important they know who to contact. | *There's all - the whole system is so complicated that you, you really don't know who to ask or what do to ... it's pretty darn confusing ... and maybe it's just our age that I get so confused.* |
|  | Receiving medical attention or guidance in a timely fashion | Patients do not have access to a medical professional in real-time. They were provided a generic phone line to the hospital or clinic where they were unable to get efficient support and this was unsettling for patients. | *Nobody can talk to you - well like this is just the receptionist, of course she can't give you any information other than to tell you the nurse will not be available for eight to ten hours. By that time, you'll be dead for sure.* |
| **Theme 3: Focusing on medical and functional recovery** | | | |
| **Sub-theme** | **Code** | **Description** | **Quotes** |
| **Sub-theme 1:** not having, or recovering from, postoperative complications | Not having complications like re-opening of incision | Patients are relieved when they do not experience complications after surgery like bleeding, incision complications or pain. | *My [post-operative transition in care was successful] because I was lucky I did not have any issues. I had no pain. I initially had constipation problems, but I took the medication and that went away. And I had no issues. That was extremely important. If I'd had pain or bleeding or, you know, like when I read this group I belong to … ladies from all over who have the same cancer, some of them go home and their, you know, their cut's open or something like that. I had none of that.* |
|  | Removal of feeding tube was a big day | Healing from postoperative complications like requiring a feeding tube for an extended period of time and experiencing weight loss is important. | *Well in my case, I lost, ah, quite a bit of weight, so stabilizing my weight loss was [important] … it was really problematic for me to start to swallow and getting all those [organ] systems back to working properly … and of course the day of freedom for me was when they took a feeding tube out of my nose. That was a big day for me. That's when I could start to swallow properly, you know? I mean as a matter of fact, my wife had to prepare almost like baby food for me, just like a newborn.* |
| **Sub-theme 2:** reclaiming independence and resuming life activities that matter | Wanting to feel normal by doing activities independently | It was important to patients to be able to participate in home activities independently to feel normal and able to participate. | *I would go down the stairs and, ah, sit downstairs for a bit just to say, "Ha! Look at me, I'm normal." And I - get this. I insisted on changing the garbage cans upstairs. I would not let my wife do that, because I had to feel worthwhile, you know? I was telling myself - and I won't let her clean my room. Um, ah - I won't let her do that. And it's a mess, but I have to have that feeling that I'm worthwhile. Like doing things like that, I know I'm going to be normal.* |
|  | Wanting to re-gain same function as before surgery | It was important to have at least the same function and mobility as before surgery but for some this was not the outcome. | *Well I wish I would ha—I was expecting to be as well as before … I cannot, ah, stand up - stand, ah, very long … Before I - I could, ah, walk let's say, to - I live at the end of, ah, the corridor and then to get to the elevator, now I find after - before, I, I could make it very easily, go and get my, my mail and things like that and down stairs and everything, but since, ah, after my operations, I couldn't do that - can't do that still ... I could—if only, if I was by myself and I had no choice, nobody to help me, then I would do it. But my walker I, I have one that I can sit on it, you know? It’s with a seat ... So I will, ah, sit—ah, sit on that. And then go with 10 minutes and then go again and, um, make another 15 minutes and then sit again. I’d say I cannot go to the shopping centre and, ah, like I used to do before my operation. Forget about that.* |
| **Sub-theme 3:** access to physiotherapy and exercise programs | Awareness and referrals to postoperative physiotherapy | Seeking out postoperative physiotherap to improve their symptoms. | *There was nothing in the [discharge pamphlet] that said anything about, um, physiotherapy. So after a while, I thought, you know? I'm stiffening up, you know? With, with 40 staples … I got a lot of scar tissue, so, so I tell people I was attacked by a shark on the Great Barrier Reef ... but I was the one, that, ah, I went out and sought, ah, physiotherapy ... and the difference it made - good lord! Because none of that mentioned in the after surgery days.* |
|  | A desire for physiotherapy or exercise instructions to improve function | Some patients wanted support and direction for how to resume activities like walking safely. | *Maybe if I could have gotten some support from physiotherapy, ah, getting down to…or getting up or whatever to physiotherapy, and them showing me exactly what I should be doing…Might have helped. Like they came and said, “Oh, you’ve got to walk. You’ve got to walk.” But never saying how long I should walk, because I used to do the walks around the—around the floor in the hospital. So I, I walk around the house. I’m up and down stairs. I’m, you know, I’m trying to do as much as I can around the house.* |
| **Theme 4: Informal caregivers and family members play multiple integral roles** | | | |
| **Sub-theme** | **Code** | **Description** | **Quotes** |
| **Sub-theme 1:** attending appointments to gather information and provide support | Support and information gathering during an overwhelming time | Some patients described the value of having someone at their appointments to help take in all of the information and provide support during such an overwhelming time. | *Um, when a person's diagnosed with cancer and you're going in for treatment or surgeries or whatever, you are walking through molasses. Time slows down … like everything slows down and you're, ah, ah, cognitive skills may not be the best … my son was with me [at the surgical consult] and I kept looking over to him going "what do you think?" ... he's a smart guy and he's in the health care field ... so I trust his judgement* |
|  | Someone attending appointments to help understand and remember all of the information | Important to have someone at appointments to help remember, digest and understand the information. | *My wife was at every meeting with [the surgeon]. It was [helpful] because it saved me trying to remember everything and tell her. Like she was right there so she, she heard everything … [the surgeon] explained everything, what happens and what it's about and he's drawing diagrams.* |
| **Sub-theme 2:** functional and practical support once home | Having someone drive to postoperative appointments | Some patients explained that they either weren’t allowed, or wouldn’t feel comfortable, driving on their own to appointments after surgery and therefore needed an informal caregiver to drive them. | *I went [for my bloodwork] with my husband. He drove me there and he came in with me and waited for my turn … I would not have been comfortable to go on my own. I, I don't know - see how - oh, because they had told me I wasn't supposed to drive … for - I don't know? Six weeks or whatever ... I would have been very worried. I don't think I would have gone by myself … I would not have driven there by myself.* |
|  | Having someone who knows you and as your interests in mind, help at home | Some informal caregivers provided support with meals and making sure the patient didn’t fall. It was important to patients to have someone who knows their preferences to be the one to help provide support. | *Well knowing that, ah, you have the right people at home [is important]. Ah, you want to be confident that you have somebody that really, ah, has your interest and some of the background at home and that you have access to all the medical care you might need … [my wife] was there to, to help me be comfortable and, she even pureed all the food and she knew what I couldn't take or could take and what I like and didn't like.*  *My husband had told me that if I had to get up and go to the bathroom, not to go by myself, wake him up and he would come with me to make sure I didn't fall.* |
| **Sub-theme 3:** social and emotional support | Needing support to get through this time | Informal caregivers help to get patients through this overwhelming time | *[My wife] made sure I got through. I shudder to think - I shudder to think - I would have just, ah, curled up … and done nothing without her support.* |
|  | Having social support and not being alone | Having family and friends phone and email was really important to not feel alone. | *Just not being alone [was important] … just to have my family support and friends calling me and, ah, or emailing me and stuff like that … that was really important. That - that to me was the biggest thing.* |
| **Theme 5: feeling supported by healthcare providers through continuity of care** | | | |
| **Sub-theme** | **Code** | **Description** | **Quotes** |
| **Sub-theme 1:** follow-up with surgeon to know if recovery is going well | Wanting to know from surgeon that they are healing well and that medical supports are working as they should be | Seeing the surgeon a few weeks after surgery was important to make sure that their medical supports were working, there were no complications or things like inflammation and they everything is going okay. | *I had regular, um, I think, every two weeks, I went back to see the surgeon or his, resident ... you know, just for check-ups and to look at the drain tube and that sort of thing. And, ah, I—I think I did three of those, up until the time when they took the drain tube out in the end of week four ... it was [important] it was because I wanted to make sure the drain tube was working properly. I mean, you—you want to be sure of that, and particularly when you have the drain tube stuck in, you want to make sure there’s no inflammation. You want to make sure it’s draining properly. Um, and that, you know, everything is—is going okay.* |
|  | Being able to ask surgeon questions about how they are feeling and what they are experiencing | It was important to see their surgeon to ask questions related to their surgery and recovery. | *Yes, it was very important seeing him because I had questions for him. No one can answer the questions—well because he knew. Questions about the pain basically. And like when I going to get rid of this drip bag?*  *I was in contact with my surgeon, you know, over those months. He phone a couple of times … I was more interested in talking to him that I was to my family doctor, actually. Because that's who I was dealing with. My family doctor, he can deal with everything else but I was dealing with the surgery and the aftermath of it, then I thought - and I'm sure my surgeon helped me out more than my family doctor could.* |
| **Sub-theme 2:** receiving postoperative homecare | Nursing care to help with specialized care | Nursing homecare provided specialized care that informal caregivers were not trained for. | *Plus, if anything went wrong at all, I had a nurse coming in every second day to check the bandages out and generally check me out … I’m not sure my wife, and certainly not me, could have handled the bandaging thing with that tube. It was a fairly specialized thing and so, the, ah, nursing service, ah, looked after that well.*  *They explained things to me [at discharge] and, ah, someone coming in to do my catheter and, ah - because I was still connected, you know, because I had to have healing time from where he did the surgery. And, ah, they - you know, I had people come in prior to going in to flush out those tubes. I had those in for two months, I think ... I knew what was going on. They came - they came in to do them … they came several times during the week.* |
|  | Homecare to support with nutrition | Some patients required more homecare support at home with died and nutrition. | The nutritionist [that came to my home] was excellent. She also was a former nurse, so she had a really good understanding of this and she provided a lot of the information that I think we were looking for … and she was very engaged … which was [important] when you're completely vulnerable, which I was ... she came once a week. Also contact by phone, so we had steady contact with her. |
| **Sub-theme 3:** receiving follow-up and having continuity of care | Follow-up with family doctor is helpful for staple removal and reassurance | It was important for patients to be seen by their family doctor or to know that they were just a phone call away if they needed anything. | *Going out to see my family doctor, I think it was three weeks to get the staples out … we - she talked to me for about 20 minutes … it felt like she just wanted to stay in touch. And she'd, you know, she'd tell me if there's any problem and you want to talk, call me at any time ... I've had her for five years now - she's always let me know if there's any problem about anything, just call, which is - it's very reassuring.* |
|  | Continuity with Psychologist | Some patients received, or desired continuity of care from professionals from disciplines such as Social Work or Psychology. | *I saw [the Psychologist] at follow-up. It was a very emotional moment … I was left with the impression the team got through this. We all got through this. Now it might not be so, ah, ah, so much attached on their end but to me, it meant everything.* |
