# Supplementary material for: “I can’t imagine having to do it on your own”: a qualitative study on postoperative transitions in care from the perspectives of older adults with frailty
Source: BMC Geriatr. 2023 Dec 13;23:848. doi: 10.1186/s12877-023-04576-9 (PMC10716948; doi:10.1186/s12877-023-04576-9)
Supplement: Supplementary file 3 — Additional file 3. [file 12877_2023_4576_MOESM3_ESM.docx]

**Supplemental Material 3 – Reflexivity Statement of E. Hladkowicz**

My role as a researcher, including my own orientations and values, aligns with those embedded in qualitative research. I suspect this is largely associated with my upbringing, my education and my ongoing curiosity about individual humans and the collective. This curiosity led me to completing my Master’s degree in Counselling Psychology where I was able to apply my innate skills with formal counselling training to understand individual lived experiences, the unique meaning that people assign to words, experiences and relationships, while considering their historical contexts and culture with the ultimate goal of supporting their healing journey. Through my counselling training, my clinical research work and my own personal relationships, I have learned, and now believe, that there can be multiple truths. I believe that each person has a unique context, with past and current experiences different from the next person, and that people have ways of thinking and seeing the world that are distinctively their own. While I believe that each person has a different perspective and experience, I also believe that there are shared thoughts, feelings and experiences across humans. This orientation and belief system is what drew me to exploring the proposed research question using a qualitative methodology.

Further, I work as a clinical research associate in a perioperative clinical research program where I have awareness of the healthcare system and clinician viewpoints. We conduct pragmatic trials and often conduct theory-driven qualitative interviews to understand barriers and facilitators to intervention implementation. Our goal is often to address real-world practice problems. I wanted to explore patient and caregiver perspectives to gain clinically relevant information to add to the postoperative transitional care literature in an applied way. Importantly, I wanted to provide a channel for older adults with frailty to share their experiences and have their voices be heard. While I have always valued the perspectives of older adults, my experience as a PhD Candidate in Aging & Health has only highlighted the need to explore patient perspectives in research.
